# The predictive role of peripheral serum inflammatory markers NLR, PLR, and LMR in ulcerative colitis and Crohn’s disease: a systematic review and meta-analysis

**DOI:** 10.3389/fimmu.2025.1623899

**Published:** 2025-07-25

**Authors:** Shufa Tan, Xiaoqing Yang, Xiaojing Mu, Shuang Liu, Yao Wang, Yuwei Li, Yuhong Bian, Chen Xu

**Affiliations:** ^1^ School of Integrative Medicine, Tianjin University of Traditional Chinese Medicine, Tianjin, China; ^2^ Tianjin Institute of Urology, the 2nd Hospital of Tianjin Medical University, Tianjin, China; ^3^ Department of Colorectal Surgery, Tianjin Union Medical Center, The First Affiliated Hospital of Nankai University, Tianjin, China

**Keywords:** inflammatory bowel disease, disease activity, NLR, PLR, LMR, meta-analysis

## Abstract

**Background:**

The neutrophil-to-lymphocyte ratio (NLR), platelet-to-lymphocyte ratio (PLR), and lymphocyte-to-monocyte ratio (LMR) are peripheral serum markers commonly utilized as cost-effective indicators of inflammation. However, their efficacy as predictors of clinical disease activity in inflammatory bowel disease (IBD), including ulcerative colitis (UC) and Crohn’s disease (CD), remains uncertain. To address this ambiguity, we conducted a meta-analysis to evaluate the clinical significance of NLR, PLR, and LMR in patients with IBD.

**Methods:**

A comprehensive search was conducted in the PubMed, Embase, Web of Science, and Cochrane databases, with the last search date being October 2024. Baseline values of NLR, PLR, and LMR during active and remission phases, as well as moderate and severe conditions, were analyzed as primary endpoints in patients with IBD compared to healthy populations, using risk ratios (WMD) and corresponding 95% confidence interval (CI) estimates.

**Results:**

Twenty-three cohort studies involving 3550 IBD patients and 1010 healthy people were finally included in this meta-analysis. The results of the meta-analysis showed that peripheral serum NLR and PLR were significantly higher in IBD patients than in the healthy population NLR [WMD=1.57,95%CI(1.14,2.01),P<0.001], PLR [WMD=60.66,95%CI(51.68,69.64),P<0.001]; NLR in active versus remission stage of IBD, PLR, LMR had significant differences NLR [WMD=1.50,95%CI(1.23,1.78),P<0.001], PLR [WMD=69.02,95%CI(39.66,98.39,P<0.001], LMR [WMD=-1.14,95%CI(-1.43,-0.86,P<0.001]; IBD active period and remission period NLR, PLR and LMR had significant differences. 0.001]; there were significant differences in NLR and PLR between moderate and severe IBD NLR [WMD=-1.41,95%CI(-2.13,-0.69),P<0.001], PLR [WMD=-112.03,95%CI(-143.87,-80.19),P<0.001]; the diagnostic accuracy of markers in predicting the clinical activity of IBD was relatively good. The diagnostic accuracy of markers in predicting IBD clinical activity was more favorable AUC [ES=0.72,95%CI(0.69,0.75),P<0.001].

**Conclusion:**

In patients with IBD, elevated NLR and PLR are associated with increased disease activity and severity in UC and CD. Conversely, an elevated LMR is linked to reduced disease activity in IBD. Based on diagnostic accuracy results, inflammatory markers NLR and PLR serve as effective biomarkers for assessing IBD activity, thereby providing valuable insights for treatment decisions in IBD patients. However, LMR may not be a reliable independent marker due to conflicting or non-significant results. We anticipate that further high-quality prospective studies will validate our findings in the future.

**Systematic review registration:**

https://www.crd.york.ac.uk/PROSPERO/, identifier CRD42024608118.

## 1 Introduction

IBD is a chronic, nonspecific inflammatory condition of the gastrointestinal tract that includes both UC and CD. UC typically presents with continuous lesions that extend from the proximal colon to the rectum and are confined to the mucosal layer. In contrast, CD can affect any part of the gastrointestinal tract, with inflammation often involving the entire bowel wall and manifesting in a patchy, segmental pattern. Both diseases share primary clinical manifestations, including recurrent abdominal pain, diarrhea, abdominal masses, mucus and bloody stools, intestinal obstruction, perforation, and weight loss. These symptoms fluctuate between episodes of relapse and remission. The exact etiology of IBD remains unclear; however, it is likely influenced by a combination of genetic, immune, environmental, and microbial factors (1). In recent years, the global incidence of inflammatory bowel disease (IBD) has been rising, with a prevalence exceeding 0.3% in North America, Oceania, and much of Europe. Currently, more than 10 million people worldwide are affected by IBD, which places a significant burden on healthcare systems and the global economy (2, 3). Although most patients achieve long-term symptom control through medication, these treatments often fail to fully suppress intestinal inflammation and are associated with various complications that substantially impact patients’ quality of life. At present, no effective cure exists for the disease (4). Furthermore, IBD is the third most significant risk factor for colorectal cancer (CRC). Studies indicate that approximately 18% of CRC cases occur in patients with IBD who have had the disease for fewer than eight years (5). With the evolving treatment paradigm for inflammatory bowel disease (IBD), achieving symptom relief and endoscopic remission has become a key therapeutic goal, which is associated with improved patient prognosis. Endoscopic cross-sectional imaging offers an accurate assessment of current intestinal inflammation and is essential for diagnosing IBD, evaluating disease severity, monitoring treatment response, and predicting relapse. However, both endoscopy and histopathology are invasive procedures that necessitate repeated colonoscopies, pathology reviews, and imaging assessments. These interventions can be costly, time-consuming, and may be hindered by low patient compliance (6). In addition, approximately one-third of patients in remission from UC experience gastrointestinal symptoms, such as abdominal pain and diarrhea, due to visceral hypersensitivity, despite the absence of disease activity on endoscopy (7). Consequently, there is an urgent need to identify non-invasive and easily accessible biomarkers for monitoring disease severity and tailoring therapeutic interventions. While white blood cell count (WBC), C-reactive protein (CRP), erythrocyte sedimentation rate (ESR), and fecal calreticulin (FC) have been proposed as clinical markers for assessing disease severity, their accuracy remains limited (8). Among the currently available markers, C-reactive protein (CRP) and erythrocyte sedimentation rate (ESR) are the most commonly used; however, they may occasionally remain normal in the presence of active inflammation (9), and their sensitivity and specificity are limited. Studies have demonstrated that the sensitivity and specificity of laboratory markers used to assess UC range from 50% to 60%, thereby limiting their clinical utility (10). In contrast, other markers, such as fecal calprotectin and lactoferrin, exhibit higher sensitivity and specificity. A meta-analysis conducted by Rokkas et al (11) reported that fecal calprotectin reflected a sensitivity of 82.4% and a specificity of 72.1% in relation to endoscopic activity in CD, with an area under the ROC curve of 0.84. Despite these promising results, fecal calprotectin has not yet achieved widespread availability due to the technical demands of the assay and its associated high costs. This situation underscores the necessity for a simpler, more user-friendly, effective, and cost-efficient biomarker capable of differentiating between quiescent and active disease states in inflammatory bowel disease (IBD), assessing mucosal recovery, predicting disease recurrence, and evaluating treatment response. They are useful in assessing the severity of many chronic diseases such as COPD (12)、hepatic encopresis (13)、rheumatoid arthritis (14)、glomerulonephritis (15) and many inflammatory disorders. Torun et al (16) observed that in patients with active ulcerative colitis (UC), elevated neutrophil-to-lymphocyte ratio (NLR) was correlated with leukocyte counts and erythrocyte sedimentation rate (ESR). Additionally, in patients with active UC, the NLR decreased significantly following the resolution of intestinal inflammation. Although numerous studies have demonstrated the potential value of peripheral blood NLR, platelet-to-lymphocyte ratio (PLR), and lymphocyte-to-monocyte ratio (LMR) in assessing the severity and progression of inflammatory bowel disease (IBD), their clinical utility remains controversial due to variations in demographic characteristics, study design, and sample size. In Cherfane’s retrospective case-control study (17), NLR effectively differentiated between active UC and controls, but not between active and inactive UC. Therefore, we conducted this meta-analysis to summarize the available evidence and explore the clinical value of peripheral serum inflammatory markers—NLR, PLR, and LMR—in patients with IBD.

## 2 Materials and methods

The protocol has been registered in the International Prospective Register of Systematic Reviews data base (PROSPERO: CRD42024608118).

### 2.1 Literature search strategy

Two researchers (TSF, YXQ) independently searched using Pubmed, Embase, Web Of Science, and Cochrane databases. Mesh words in PubMed were used to broaden the search, and search terms included “UC”, “ulcerative colitis”, “CD”, “Crohn’s disease”, “IBD”, “Inflammatory Bowel Disease”, “Neutrophil to lymphocyte ratio”, “NLR”, “Platelet to lymphocyte ratio”, “PLR”, “Lymphocyte to monocyte ratio”, “LMR”. The search formula is: ((((((ulcerative colitis) OR (Crohn’s disease)) OR (Inflammatory Bowel Disease)) OR (UC)) OR (CD)) OR (IBD)) AND ((((((Neutrophil to lymphocyte ratio) OR (Platelet to lymphocyte ratio)) OR (Lymphocyte to monocyte ratio)) OR (NLR)) OR (PLR)) OR (LMR)). The search strategy did not restrict language or study type, and the search time frame was from 2000 to October 2024. Two researchers screened the articles based on the title, abstract, and inclusion/exclusion criteria. The two researchers performed the extraction and review of data on basic information of relevant literature, study objectives, outcomes and follow-up, and in case of disagreement, the data were judged by third-party experts. Systematic evaluation was performed according to the Preferred Reporting Items for Systematic Evaluation and Meta-Analysis (PRISMA) guidelines (18).

### 2.2 Inclusion and exclusion criteria

Inclusion criteria were as follows:

1. Clinically confirmed diagnosis of inflammatory bowel disease (ulcerative colitis, Crohn’s disease).
2. Studies reporting the expression of inflammatory markers NLR, PLR, and LMR in different periods of IBD using risk ratios (WMD) and 95% confidence intervals (CI).
3. Grouping according to exposure into exposed group (high expression of inflammatory markers) and non-exposed group (low expression of inflammatory markers).
4. Literature in both Chinese and English.
5. Endpoint indicators were expression of markers in IBD patients versus healthy population, expression in active versus remission disease, expression in moderate versus severe disease, and diagnostic accuracy AUC.
6. Included study designs were randomized controlled trials, observational studies, cross-sectional studies, retrospective studies or prospective studies.

The exclusion criteria were as follows:

1. Disease study type or intervention approach not met.
2. Information on outcome indicators was not available.
3. Endpoint indicators could not be extracted.
4. Duplicate publications or incomplete information.
5. Non-comparative studies, animal experiments, reviews, letters, guidelines, case reports, pathomechanisms, conference abstracts, expert opinions, editorials, commentaries.
6. Literature in other languages.

### 2.3 Data extraction

Two researchers independently screened the literature according to the inclusion and exclusion criteria, information was independently extracted using a standardized data extraction form, cross-checked individually by the two researchers, and disagreements were resolved through discussion. Studies were excluded if relevant data were not available. For each study, the following information was collected: (1) study characteristics: first author, country, year of publication, type of study, type of IBD, type of predictor, duration of disease, cut-off value, diagnostic criteria; (2) patient baseline: number of patients, age, gender; (3) study outcomes: expression of markers in IBD patients versus healthy populations, and expression of markers in active versus remission phases of disease, expression of moderate versus severe disease, and diagnostic accuracy AUC.

### 2.4 Literature quality assessment

The quality of the included cohort studies was independently assessed using the Newcastle-OttawaScale (NOS), which consists of three metrics: cohort selection, comparability, and outcome assessment. The modified NOS is a 9-point scale, with low quality studies scoring 1-3, moderate quality 4-6, and high quality 7-9. Scoring was done independently by two investigators, and third-party experts were consulted to resolve any large differences between their scores or if this affected the study’s inclusion in the final analysis.

### 2.5 Statistic analysis

StataSE16.0 software was used for statistical analysis to calculate the combined WMD and 95% confidence intervals (95% CI), and P<0.05 showed a significant difference between the two groups. Heterogeneity was evaluated using I² values,I²≤30%,30%<I²<75% and ≥75% were considered to indicate low, medium and high heterogeneity, respectively.I²<50% was analyzed using a fixed-effects model, while I²≥50% was analyzed using a random-effects model. Sensitivity analyses were performed for outcomes with high heterogeneity, excluding a study in the merger one by one, evaluating the combined effect values and changes in heterogeneity in the remaining literature, and analyzing the sources of heterogeneity, and assessing whether there was publication bias using Begg’s funnel plot and Egger’s test, with P > 0.05 indicating that there was no publication bias.

## 3 Results

### 3.1 Literature search results

**Figure 1:**
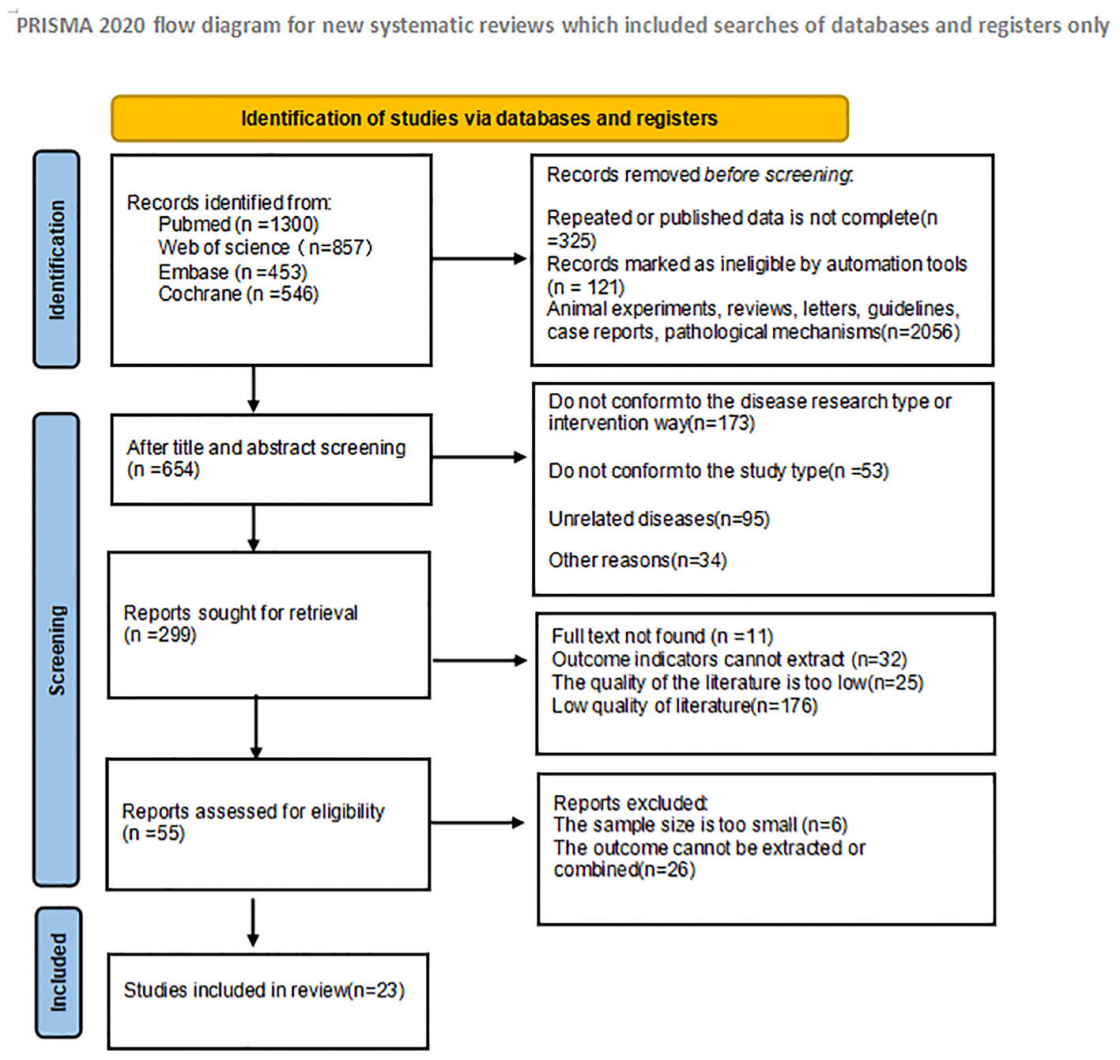
Schematic diagram of literature search criteria and including studies in meta-analyses.

In the initial literature search, a total of 3156 articles were searched. 325 duplicate studies were excluded; After reading the title and abstract of the article, 2776 studies were excluded according to the inclusion criteria, and 55 studies were initially included. We then read the full text and excluded 32 studies that did not meet the inclusion criteria. Finally, 23 studies were included in the meta-analysis. The literature screening process and results are shown in **Figure 1**:

### 3.2 Basic characteristics of the included studies

As shown in **Table 1**, among the 23 studies included, 3550 patients with IBD and 1010 healthy people were involved. All 23 studies were cohort studies, including 18 retrospective cohort studies and 5 prospective cohort studies. Multiple inflammatory markers were studied in one study in the included literature, so we numbered different inflammatory markers in the same literature. Study characteristics, patient baseline, and study results of the included studies are shown in **Table 1**.

**Table 1:** Clinical and demographic characteristics of studies included in the meta-analysis.

First author	Year	Multicenter/Monocentric	Research type	Author states	IBD type	Sample size		Sex (Male/female)		Baseline		Duration(years)	Cut-off	Diagnostic criteria	Type	Outcomes
						Patients	Control Group	Patients	Control Group	Patients	Control Group					
Tian Liu (19)	2024	monocentric	retrospective	China	UC	81	77	49/32	42/35	2.48(1.64–4.01)	1.55(1.24–1.94)	3(0.16–8)	3.46	Mayo score	NLR	1、2、3、4
Masashi Omori (20)	2024	multicenter	retrospective	Japan	UC	47	NA	27/20	NA	NA		5 ± 8.9	3.59	Lichtiger index	LMR	2
Pınar Şimşek-Onat (21)	2023	monocentric	retrospective	Turkey	UC	22	92	NA	NA	2.22 ± 1.29	0.92 ± 0.32	NA	2.14	PUCAI	NLR	1、2、4
Pınar Şimşek-Onat2 (21)	2023	monocentric	retrospective	Turkey	CD	39	92	NA	NA	3.12 ± 0.99	0.92 ± 0.32	NA		PCDAI	NLR	5
Noriyuki Kurimoto (22)	2023	monocentric	retrospective	Japan	UC	129	NA	67/62	NA	1.98 (1.39–2.59)	NA	12.3 (6.8–18.8)	NA	Mayo score	NLR	2、4
Wan Feng (23)	2022	monocentric	retrospective	China	UC	306	NA	169/137	NA	NA		4(1-7)	2.19	Truelove	NLR	2、4
Wan Feng2 (23)	2022	monocentric	retrospective	China	UC	306	NA	169/137	NA	NA		4(1-7)	147.96	Truelove	PLR	3
Jiawei Cui (24)	2022	monocentric	retrospective	China	UC	386	NA	219/167	NA	2.19 (1.67)	NA	3 ± 5.25	1.97	Mayo score	NLR	3
Jiawei Cui2 (24)	2022	monocentric	retrospective	China	UC	386	NA	219/167	NA	163.60 (104.71)	NA	3 ± 5.25	145.66	Mayo score	PLR	3
Jiawei Cui3 (24)	2022	monocentric	retrospective	China	UC	386	NA	219/167	NA	3.52 (2.17)	NA	3 ± 5.25	3.92	Mayo score	LMR	2、4
Sami Cifci (25)	2021	monocentric	retrospective	Turkey	UC	165	NA	100/65	NA	NA		NA	2.06	Rachmilewitz	NLR	1、3、4
Yujin Jeong (26)	2021	monocentric	retrospective	Korea	UC	48	96	26/22	NA	3.24 ± 2.78	1.52 ± 0.61	NA	2.26	Mayo score	NLR	1、3、4
Yujin Jeong2 (26)	2021	monocentric	retrospective	Korea	UC	48	96	26/22	NA	187.01 ± 136.94	132.88 ± 45.72	NA	179.8	Mayo score	PLR	2
Katsuya Endo (27)	2021	monocentric	retrospective	Japan	UC	48	NA	29/19	NA	3.55 (2.48–7.04)	NA	0.5 (0–3)	3.6	Mayo score	NLR	2
Katsuya Endo2 (27)	2021	monocentric	retrospective	Japan	UC	48	NA	29/19	NA	207.2 (174.4–243.6)	NA	0.5 (0–3)	262	Mayo score	PLR	2、5
Yu Nishida (28)	2019	monocentric	retrospective	Japan	UC	45	NA	26/19	NA	5.84 (3.25–9.45)	NA	2.7 (1.2–8.6)	NA	Mayo score	NLR	1、2、3
Ashraf M. Okba (29)	2019	monocentric	retrospective	Egypt	UC	80	40	34/46	28/12	2.63 ± 0.43	1.44 ± 0.19	NA	1.91	Mayo score	NLR	1、2、3
Ashraf M. Okba2 (29)	2019	monocentric	retrospective	Egypt	UC	80	40	34/46	28/12	3.64 ± 0.49	2.25 ± 0.51	NA	2.88	Mayo score	LMR	1、2
Muhammet Yener (30)	2018	monocentric	retrospective	Turkey	UC	104	105	45/59	47/58	2.9 ± 0.8	1.8 ± 0.6	NA	NA	Rachmilewitz	NLR	1、2
Muhammet Yener2 (30)	2018	monocentric	retrospective	Turkey	UC	104	105	45/59	47/58	153.7 ± 72.1	104.1 ± 30.4	NA	NA	Rachmilewitz	PLR	2
Cynthia E. Cherfane (17)	2015	monocentric	retrospective	USA	UC	110	75	55/55	33/42	NA	NA	4(2–9)	NA	Mayo score	NLR	2
Cynthia E. Cherfane2 (17)	2015	monocentric	retrospective	USA	UC	110	75	55/55	33/42	NA	NA	4(2–9)	NA	Mayo score	LMR	1、2、4
Ayse Kevser Demir (31)	2015	monocentric	retrospective	Turkey	UC	71	140	47/24	84/56	2.59 ± 1.47	1.98 ± 0.85	4.75 ± 2.02	2.39	Truelove	NLR	2、4
Emrah Posul (32)	2014	monocentric	retrospective	Turkey	UC	49	NA	27/22	NA	NA	NA	3.0 (1.2–7.0)	2.3	MTWSI	NLR	1、2、3
Mehmet Celikbilek (33)	2013	monocentric	retrospective	Turkey	UC	28	26	18/8	10/18	3.18 ± 1.76	1.77 ± 0.68	2.75(1.03–6.00)	2.47	MTWSI	NLR	1、2
Serkan Torun (16)	2012	monocentric	retrospective	Turkey	UC	196	59	125/71	34/25	2.67 ± 1.29	2.01 ± 0.64	5(0.33-25)	2.16	Truelove	NLR	2、4
Yi-Han Chen (34)	2020	monocentric	retrospective	China	UC	275	NA	151/124	NA	NA	NA	1.31(0.5-4)	2.4	Mayo score	NLR	2、4
Yi-Han Chen2 (34)	2020	monocentric	retrospective	China	UC	275	NA	151/124	NA	NA	NA	1.31(0.5-4)	187.68	Mayo score	PLR	2、4
Yi-Han Chen3 (34)	2020	monocentric	retrospective	China	UC	275	NA	151/124	NA	NA	NA	1.31(0.5-4)	3.56	Mayo score	LMR	2、4
Yi-Han Chen4 (34)	2020	monocentric	retrospective	China	CD	601	NA	438/163	NA	NA	NA	1.83(0.61-3.65)	3.32	Mayo score	NLR	2、4
Yi-Han Chen5 (34)	2020	monocentric	retrospective	China	CD	601	NA	438/163	NA	NA	NA	1.83(0.61-3.65)	191.22	Mayo score	PLR	2、4
Yi-Han Chen6 (34)	2020	monocentric	retrospective	China	CD	601	NA	438/163	NA	NA	NA	1.83(0.61-3.65)	2.67	Mayo score	LMR	2、4
Mengque Xu (35)	2019	monocentric	prospective	China	UC	73	NA	42/31	NA	NA	NA	NA	NA	Truelove	NLR	2
Mengque Xu2 (35)	2019	monocentric	prospective	China	UC	73	NA	42/31	NA	NA	NA	NA	NA	Truelove	LMR	2
Mengque Xu3 (35)	2019	monocentric	prospective	China	CD	141	NA	104/37	NA	NA	NA	NA	NA	Truelove	NLR	2
Mengque Xu4 (35)	2019	monocentric	prospective	China	CD	141	NA	104/37	NA	NA	NA	NA	NA	Truelove	LMR	2
Jue-Rong Feng (36)	2017	monocentric	prospective	China	CD	103	87	72/31	65/22	2.95 (0.23–46.85)	2.33 (0.95–10.17)	NA	2.72	Mayo score	NLR	1
Jue-Rong Feng2 (36)	2017	monocentric	prospective	China	CD	103	87	72/31	65/22	3.02 ± 1.91	4.1 ± 1.31	NA	6.57	Mayo score	LMR	1
Jue-Rong Feng3 (36)	2017	monocentric	prospective	China	CD	103	87	72/31	65/22	171.61 (54.44–850)	93.49 (34.23–500)	NA	132.88	Mayo score	PLR	1
Meng-Hui Zhang (37)	2021	monocentric	prospective	China	UC	172	172	91/81	96/76	4.31 (2.73-6.47)	1.83 (1.45-2.47)	NA	2.66	Mayo score	NLR	1
Meng-Hui Zhang2 (37)	2021	monocentric	prospective	China	UC	172	172	91/81	96/76	191.87 (130.97-263.89)	127.49 (98.71-156.21)	NA	156.54	Mayo score	PLR	1
Amr Shaaban Hanafy (38)	2018	monocentric	prospective	Egypt	UC	168	NA	100/68	NA	NA	NA	NA	2.35	Truelove	NLR	2
Acarturk G (39)	2015	monocentric	prospective	Turkey	UC	42	41	28/14	14/27	4.12 ± 1.41	1.68 ± 0.59	NA	3.1	Truelove	NLR	1、2
Acarturk G2 (39)	2015	monocentric	prospective	Turkey	CD	21	41	12/9	14/27	5.25 ± 1.85	1.68 ± 0.59	NA	3.2	Truelove	NLR	1、2

(1: Comparison of marker levels in IBD and healthy people 2: comparison of marker levels in activity and remission 3: comparison of marker levels in moderate and severe 4: diagnostic performance AUC 5: clinical recurrence; NLR, Neutrophil-lymphocyteratio; PLR, Platelet-lymphocyteratio; LMR, lymphocyte-monocyteratio; UC, ulcerative colitis; CD, Crohn’s disease; NA, Not mentioned in the original article).

### 3.3 The quality assessment of the included studies

The quality of the included cohort studies was evaluate during the Newcastle-OttawaScale(NOS) for quality and the overall quality was rate dasgood, with the results shown in **Table 2**.

**Table 2:** NOS quality evaluation table.

Study	Selection	Comparability	Outcomes	Total
	1234		123	
Tian Liu	★★★	★★	★★	7
Masashi Omori	★★★	★	★★★	7
Pınar Şimşek−Onat	★★★	★	★★★	7
Noriyuki Kurimoto	★★★	★★	★★	7
Wan Feng	★★★	★★	★★★	8
Jiawei Cui	★★★	★	★★★	7
Sami Cifci	★★★	★★	★★★	8
Yujin Jeong	★★★	★	★★	6
Katsuya Endo	★★★	★★	★★★	8
Yu Nishida	★★★	★	★★★	7
Ashraf M. Okba	★★★	★★	★★	7
Muhammet Yener	★★★	★★	★★★	8
Cynthia E. Cherfane	★★★	★	★★★	7
Ayse Kevser Demir	★★★	★★	★★★	8
Tanja Mesti	★★	★★	★★★	7
Emrah Posul	★★★	★★	★★	7
Mehmet Celikbilek	★★★	★	★★★	7
Serkan Torun	★★	★	★★★	6
Yi-Han Chen	★★★	★	★★★	7
Mengque Xu	★★★	★★	★★★	8
Jue-Rong Feng	★★★	★★	★★★	8
Meng-Hui Zhang	★★★	★	★★★	7
Amr Shaaban Hanafy	★★★	★	★★★	7
Acarturk G	★★★	★★	★★★	8

(★ represents the score, and one ★ is one point).

### 3.4 Meta-analysis results

#### 3.4.1 IBD and NLR levels in healthy people

**Figure 2:**
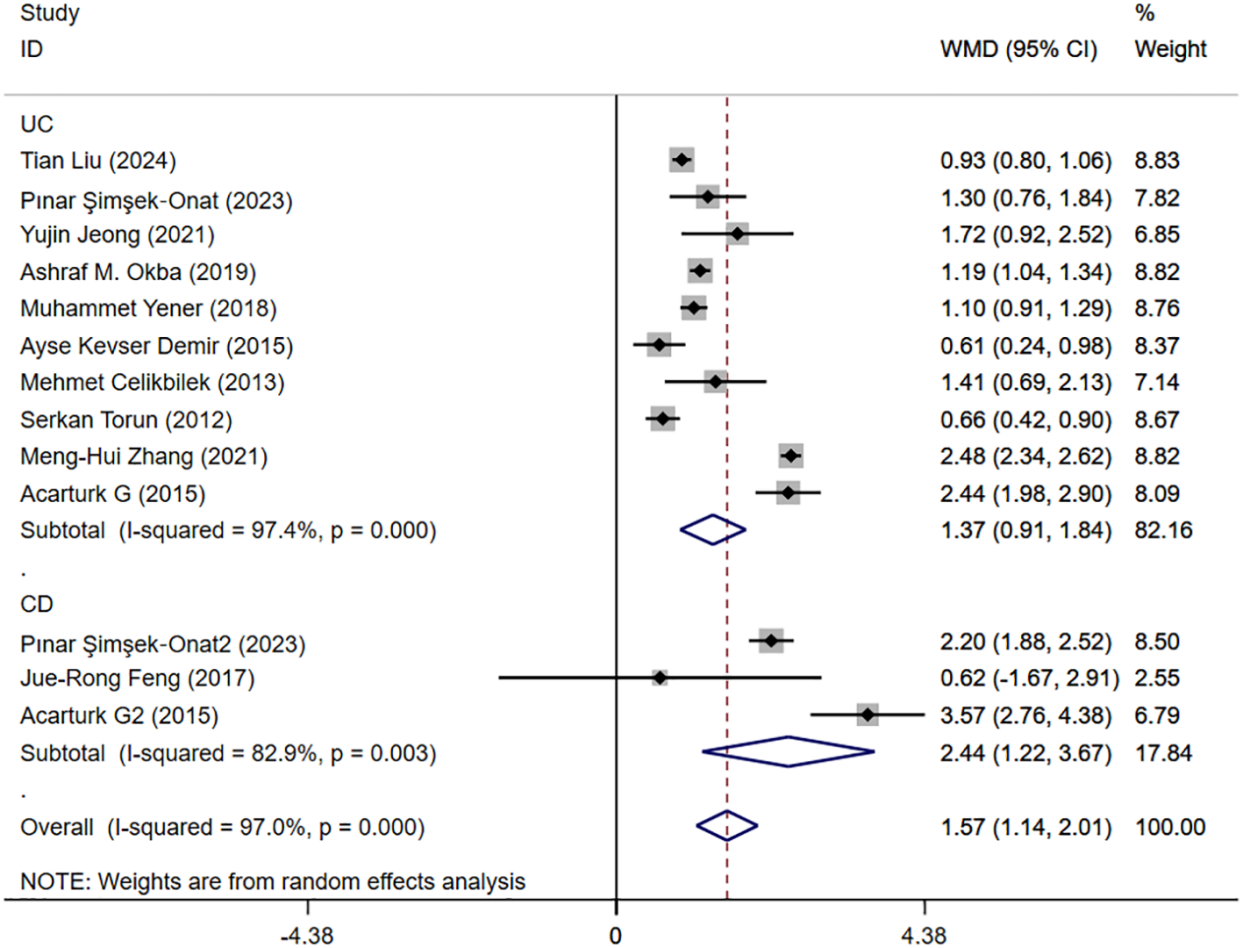
The study’s forest maps evaluated NLR levels in IBD patients versus healthy people (CD, Crohn’s disease; UC, ulcerative colitis; WMD, standard mean difference).

**Figure 3:**
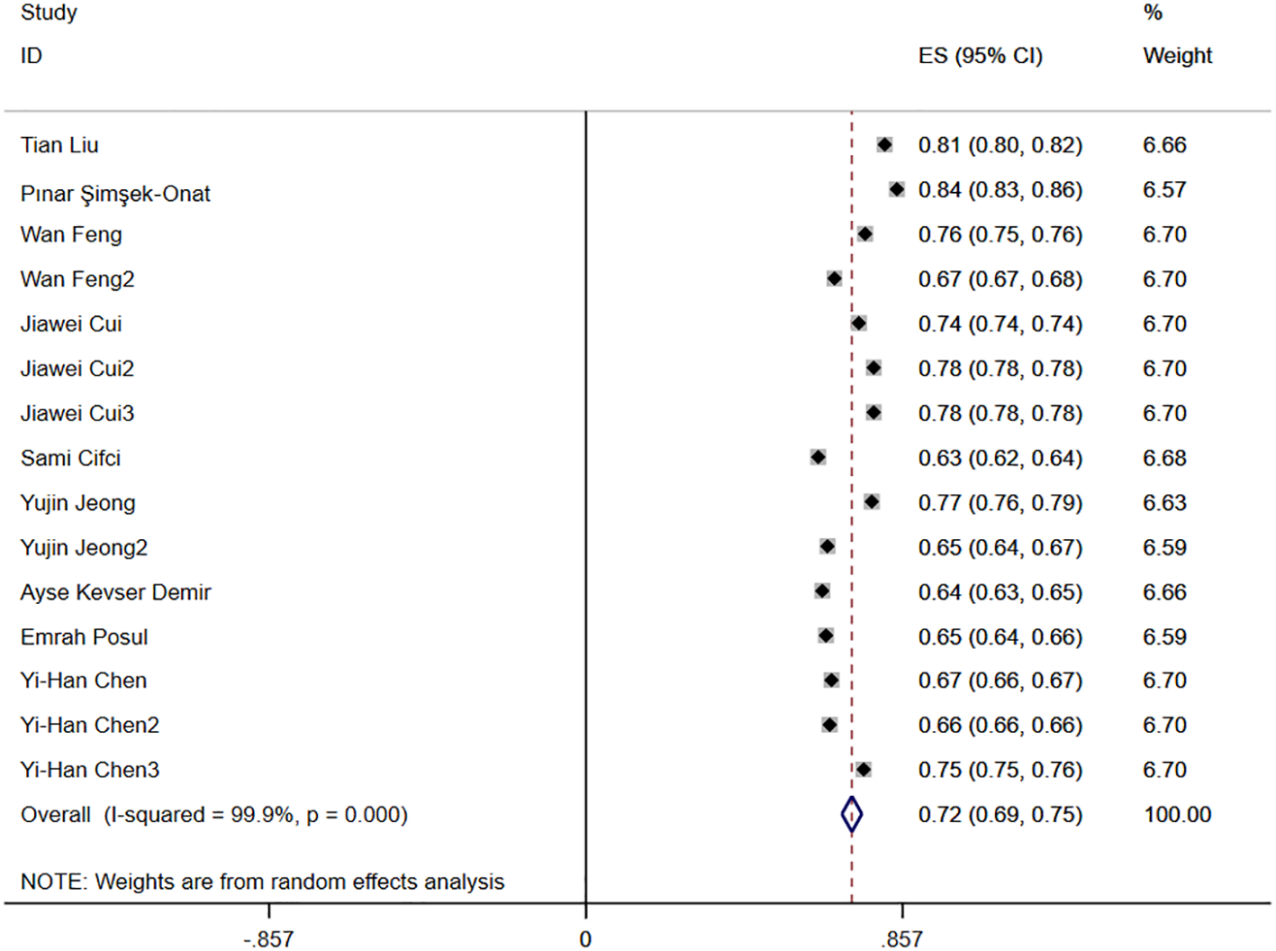
Merged AUC forest map.

IBD and NLR levels in healthy people were reported in 11 studies. **Figure 2** shows the risk-ratio forest plots identified in 11 studies. Considering the large heterogeneity between studies (P < 0.001,I²=97%), a random effects model was used for meta-analysis. Analysis results showed that high levels of NLR were significantly expressed in both UC and CD patients: UC[WMD=1.37,95%CI(0.91,1.84),P < 0.001], CD[WMD=2.44,95%CI(1.22,3.67),P < 0.001]. Considering the existence of large heterogeneity, sensitivity analysis was performed. When each study was excluded in turn, there was no substantial change in the aggregated WMD (**Figure 3**). Therefore, subgroup analysis and regression analysis were conducted to explore the sources of heterogeneity (**Tables 3**, **4**).

**Table 3:** NLR merges for subgroup analysis.

NLR									
Subgroup	IBD VS Health				Active VS Relieve				
	Study	WMD [95%CI]	P value	I^2^		Study	WMD [95%CI]	P value	I^2^
Country									
China	3	1.50 (0.14,2.86)	P<0.001	99.2	China	6	1.37 (0.95,1.80)	P<0.001	96.9
Turkey	8	1.61 (1.07,2.16)	P<0.001	94.5	Turkey	10	1.49 (0.98,2.00)	P<0.001	93.9
Korea	1	1.72 (0.92,2.52)	P<0.001	NA	Japan	2	2.51 (0.81,4.22)	P=0.004	90.5
Egypt	1	1.19 (1.04,1.34)	P<0.001	NA	Egypt	2	1.68 (0.30,3.06)	P=0.017	97.1
					USA	1	0.64 (0.52,0.76)	P<0.001	NA
IBD Type									
UC	10	1.37 (0.91,1.84)	P<0.001	97.4	UC	17	1.31 (1.07,1.56)	P<0.001	94.4
CD	3	1.93 (1.49,2.49)	P=0.003	82.9	CD	4	2.41 (1.52,3.29)	P<0.001	81.8
Sample size									
<100	9	1.64 (1.23,2.06)	P<0.001	93.6	<100	13	1.79 (1.30,2.27)	P<0.001	94.8
≥100	4	1.32 (0.25,2.38)	P=0.016	98.6	≥100	8	1.22 (0.84,1.61)	P<0.001	97.8
Diagnostic criteria									
Mayo score	5	1.50 (0.71,2.28)	P<0.001	98.5	Mayo score	7	1.51 (1.02,1.99)	P<0.001	98.3
Lichtiger index	1	1.30 (0.76,1.84)	P<0.001	NA	PUCAI	1	3.07 (1.48,4.66)	P<0.001	NA
PUCAI	1	2.20 (1.88,2.52)	P<0.001	NA	PCDAI	1	2.65 (1.57,3.73)	P<0.001	NA
Rachmilewitz	1	1.10 (0.91,1.29)	P<0.001	NA	Truelove	8	1.82 (1.28,2.37)	P<0.001	93.1
Truelove	4	1.77 (0.67,2.86)	P<0.001	96.5	Rachmilewitz	2	0.68 (0.46,0.91)	P<0.001	0
MTWSI	1	1.41 (0.69,2.13)	P<0.001	NA	MTWSI	2	0.60 (0.46,0.73)	P<0.001	0
Study design									
Retrospective	9	1.19 (0.92,1.46)	P<0.001	89.5	Retrospective	16	1.27 (0.98,1.55)	P<0.001	96.7
Prospective	4	2.61 (2.11,3.11)	P=0.024	68.1	Prospective	5	2.56 (2.08,3.05)	P<0.001	51.2

**Table 4:** Meta-regression analysis.

Covariate	Univariable					Multivariable				
Subgroup	Coefficients	Lower bound	Upper bound	Std. error	p-Value	Coefficients	Lower bound	Upper bound	Std. error	p-Value
Patients	0.99	0.99	1.00	0.002	0.761	0.99	0.99	1.00	0.001	0.419
IBD type	2.06	0.97	4.35	0.001	0.057	1.48	0.71	3.11	0.515	0.273
Research type	2.32	1.20	4.48	0.72	0.014	2.61	1.33	5.08	0.817	0.008
Country	0.96	0.74	1.25	0.12	0.81	0.89	0.72	1.10	0.088	0.287
Diagnostic criteria	0.91	0.77	1.06	0.069	0.20	0.85	0.74	0.97	0.054	0.027

#### 3.4.2 Activity and remission NLR levels in patients with IBD

**Figure 4:**
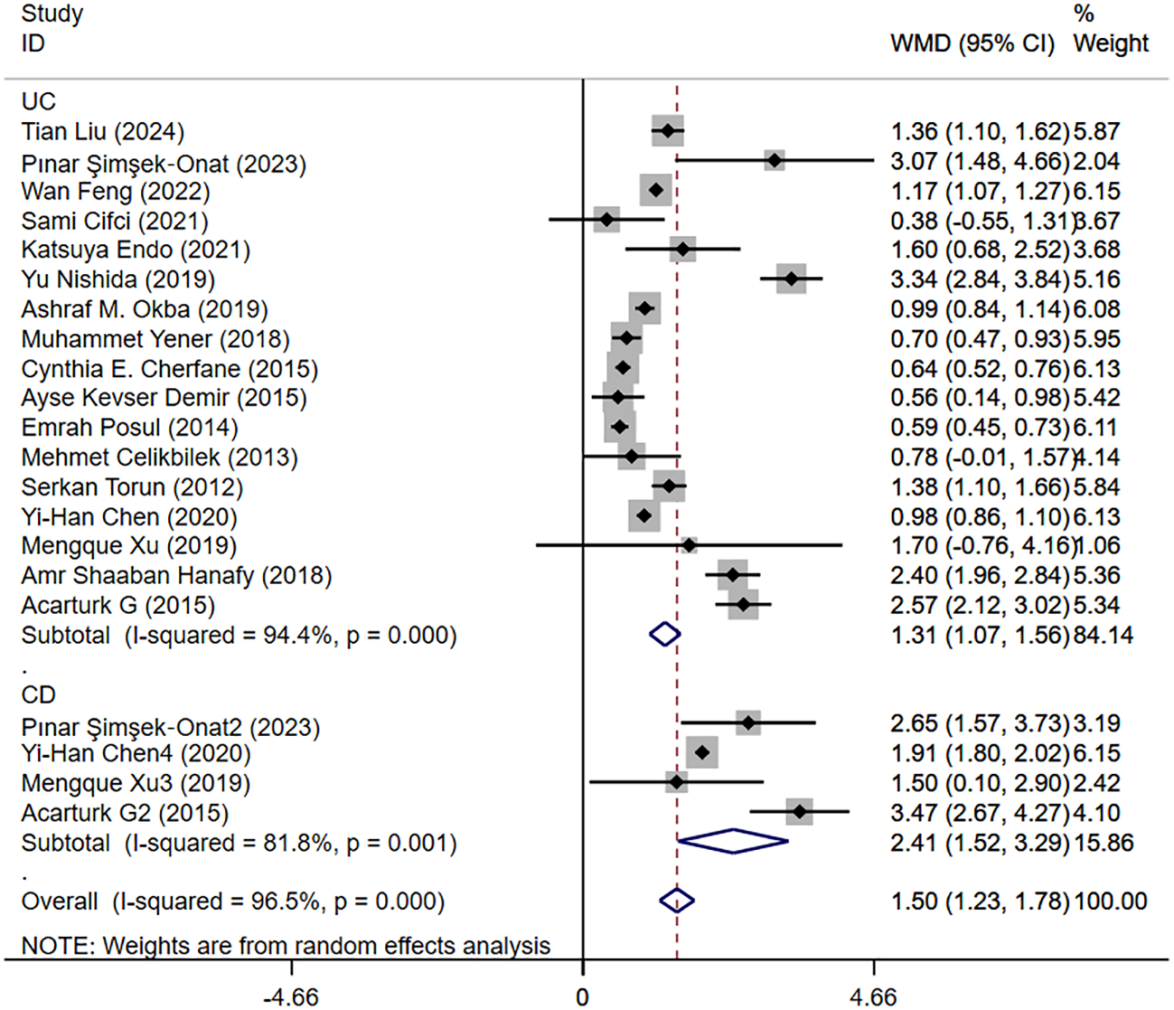
The study’s forest map assessed activity and remission NLR levels in patients with IBD (CD, Crohn’s disease; UC, ulcerative colitis; WMD, standard mean difference).

**Figure 5:**
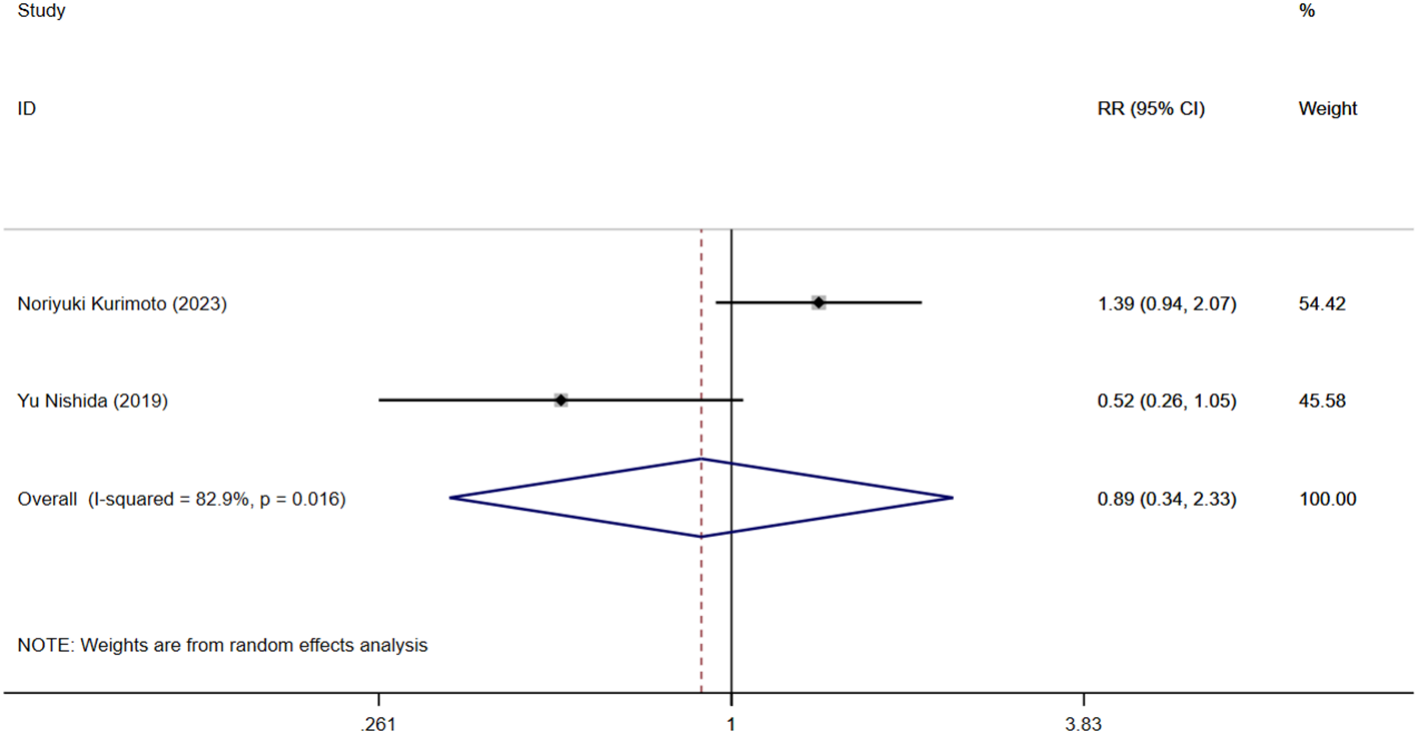
Forest map of NLR levels against recurrence rates.

Seventeen studies reported activity and remission NLR levels in patients with IBD. **Figure 4** shows the risk-ratio forest plots identified in 17 studies. Considering the large heterogeneity between studies (P < 0.01,I²=96.5%), a random effects model was used for meta-analysis. Analysis results showed that serum NLR levels in active IBD patients were significantly higher than those in remission: UC[WMD=1.31,95%CI(1.07,1.56),P < 0.001], CD[WMD=2.41,95%CI(1.52,3.29),P < 0.001]. Considering the existence of large heterogeneity, sensitivity analysis was performed. When each study was excluded in turn, there was no substantial change in the aggregated WMD (**Figure 5**). Therefore, subgroup analysis and regression analysis were conducted to explore the sources of heterogeneity (**Tables 3**, **4**).

#### 3.4.3 Moderate and severe NLR levels in patients with IBD

**Figure 6:**
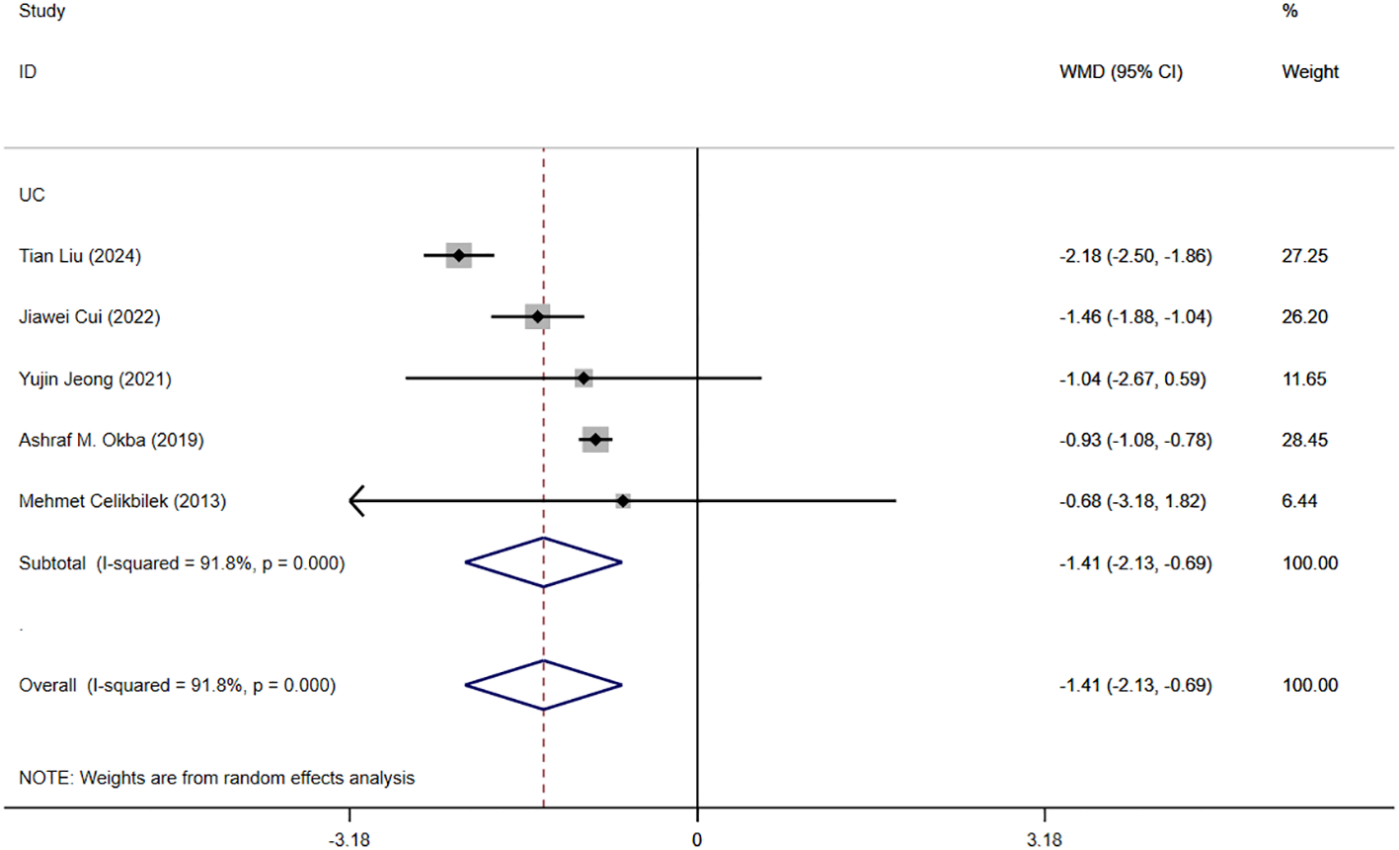
The study’s forest map assessed severe and moderate NLR levels in patients with IBD (CD, Crohn’s disease; UC, ulcerative colitis; WMD, standard mean difference).

Five studies reported moderate and severe NLR levels in patients with IBD. **Figure 6** shows the risk-ratio forest plots identified in the five studies. Considering the large heterogeneity between the studies (P < 0.001,I²=91.8%), a random effects model was used for meta-analysis. Analysis results showed that the severity of UC patients was positively correlated with the level of NLR: [WMD=-1.41,95%CI(-2.13,-0.69), P < 0.001].

#### 3.4.4 PLR levels in IBD patients

**Figure 7:**
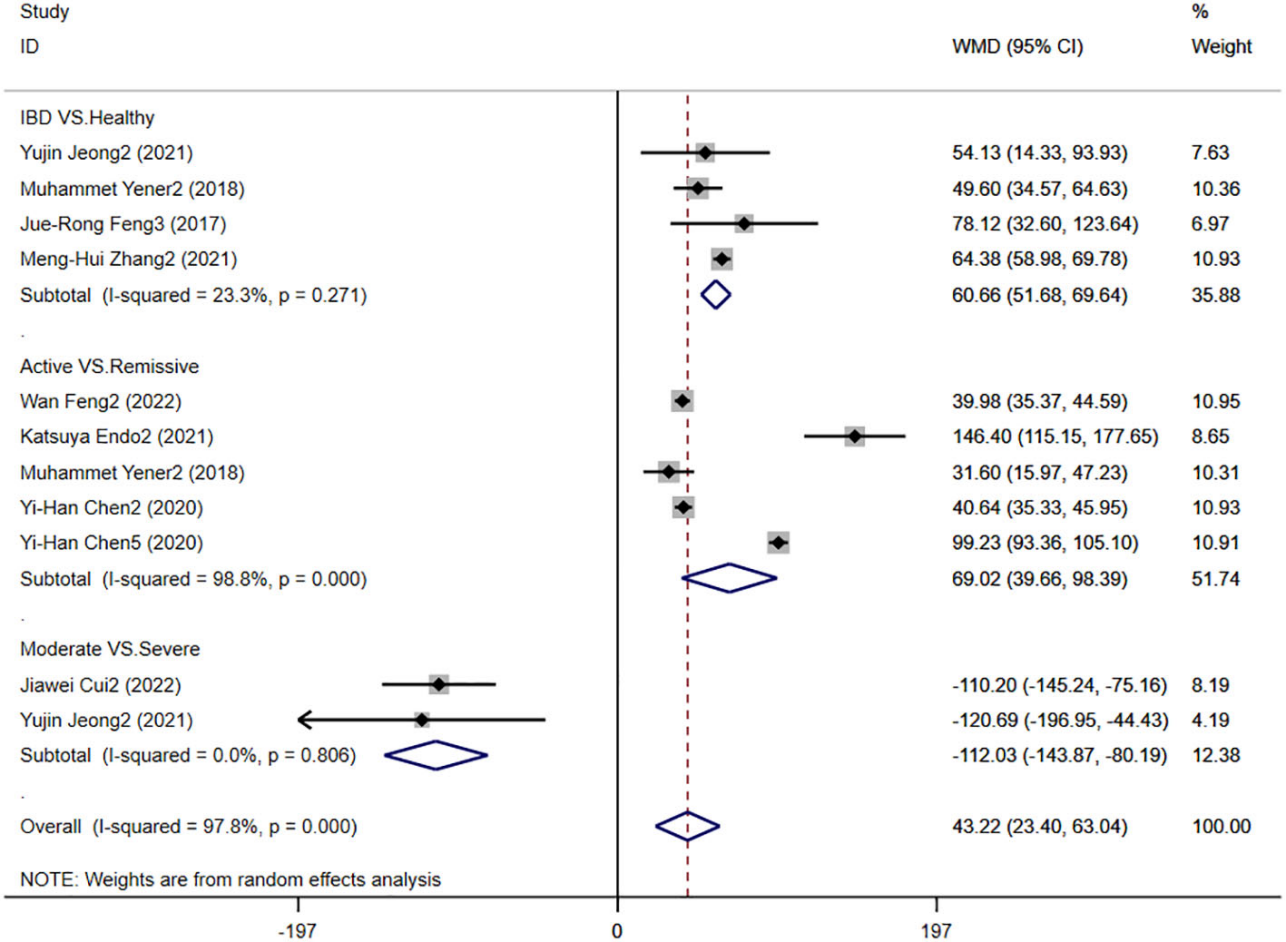
The study’s forest map assessed PLR levels in patients with IBD (CD, Crohn’s disease; UC, ulcerative colitis; WMD, standard mean difference).

Eight studies reported PLR levels in patients with IBD, and **Figure 7** shows the risk-ratio forest plots identified in the eight studies, using a random effects model for meta-analysis. The results showed that there were significant differences in serum PLR between IBD patients and healthy people [WMD=60.66,95%CI(51.68,69.64),P < 0.001]. The serum PLR level of IBD patients in active stage was significantly higher than that in remission stage [WMD=69.02,95%CI(39.66,98.39),P < 0.001]. The severity of IBD was positively correlated with PLR level [WMD=-112.03,95%CI(-143.87,-80.19), P < 0.001].

#### 3.4.5 LMR levels in patients with IBD

**Figure 8:**
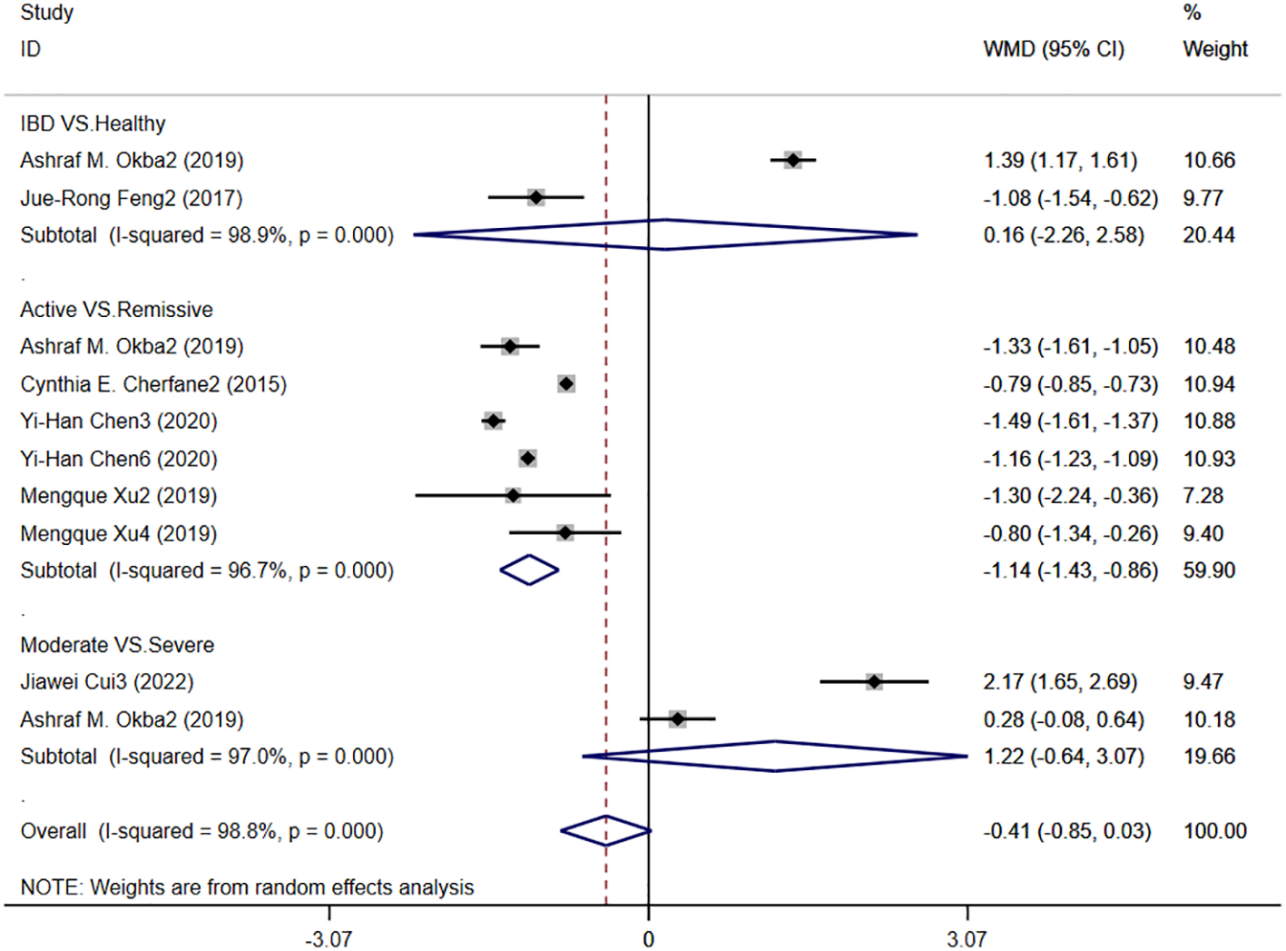
The study’s forest map assessed LMR levels in patients with IBD (CD, Crohn’s disease; UC, ulcerative colitis; WMD, standard mean difference).

Six studies reported LMR levels in patients with IBD, and **Figure 8** shows the hazard ratio forest plot determined in the six studies, using a random effects model for meta-analysis. The results showed that there was no significant difference in serum LMR between IBD patients and healthy people [WMD=0.16,95%CI(-2.26,69.2.58), P < 0.001]. The serum LMR level of IBD patients in active stage was significantly lower than that in remission stage [WMD=-1.14,95%CI(-1.43,-0.86),P < 0.001]. There was no significant difference between LMR level and disease severity in IBD patients [WMD=1.22,95%CI(-0.64,3.07), P < 0.001].

#### 3.4.6 AUC

Nine studies reported the diagnostic accuracy of NLR, and **Figure 3** shows the hazard ratio forest plots determined in the nine studies, using a random effects model for meta-analysis. Analysis results showed that NLR had good accuracy in diagnosing IBD [ES=0.72,95%CI(0.69,0.75), P < 0.001].

#### 3.4.7 Clinical recurrence

Two studies reported that NLR levels were associated with clinical recurrence of IBD. **Figure 5** shows the risk-ratio forest plots determined by the two studies. Meta-analysis was conducted using a random effects model. The results showed that high level of NLR and low level of NLR had no statistical significance on IBD recurrence rate [RR=0.892,95%CI(0.342,2.327), P = 0.815].

### 3.5 Sensitive analysis

**Figure 9:**
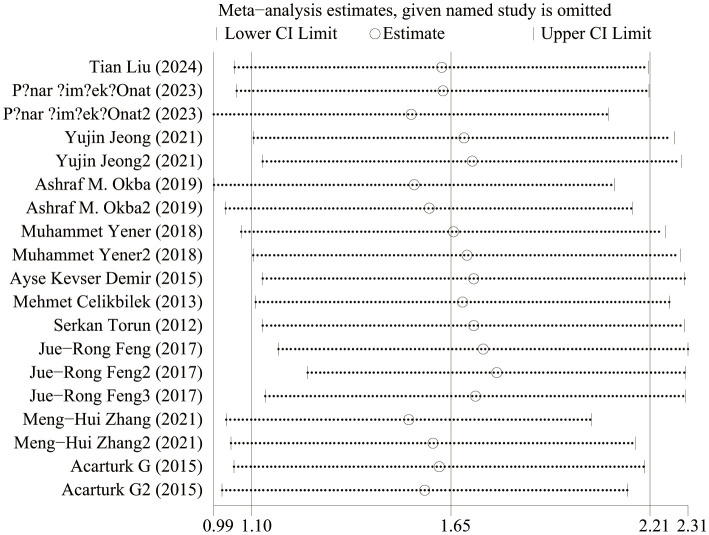
Sensitivity analysis of NLR levels in IBD patients and healthy people.

**Figure 10:**
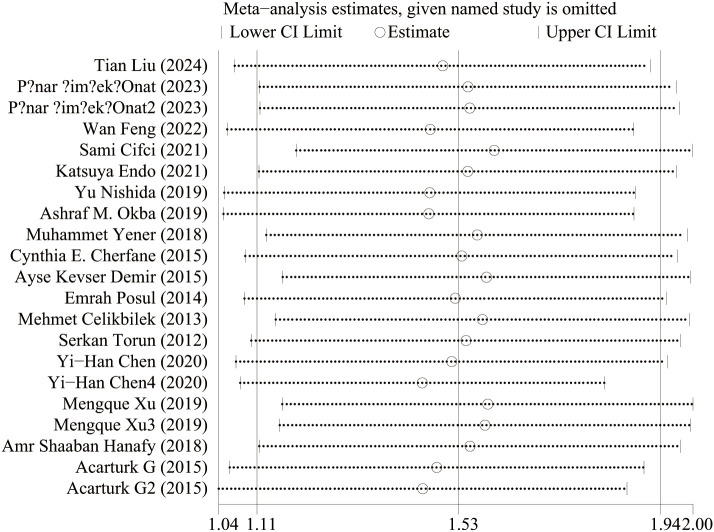
Sensitivity analysis of activity and remission NLR levels in IBD patients.

**Figure 9** shows the sensitivity analysis of NLR levels in IBD patients and healthy people. When each study was excluded in turn, the aggregated WMD did not change substantially, and the model was robust and reliable. **Figure 10** shows the sensitivity analysis of IBD patients’ activity and NLR levels in remission. When each study was excluded in turn, the aggregated WMD did not change substantially, indicating that the model was robust and reliable.

### 3.6 Publication bias

**Figure 11:**
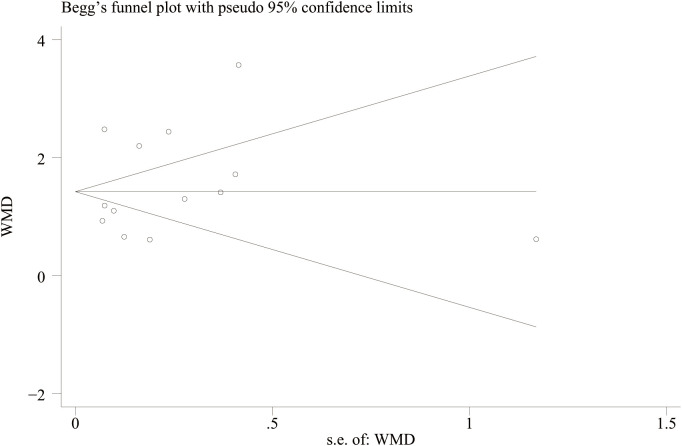
Funnel plot for the evaluation of publication bias for NLR levels in IBD patients and healthy people.

**Figure 12:**
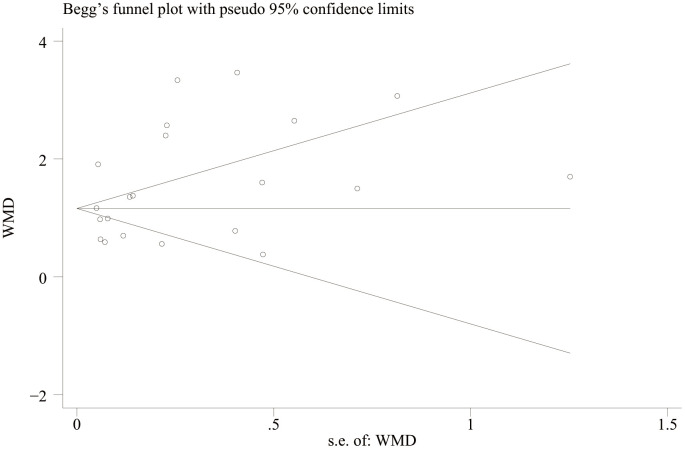
Funnel plot for the evaluation of publication bias for activity and remission NLR levels in IBD patients.

A funnel plot was drawn to evaluate publication bias between the NLR level of IBD patients and healthy people and between the NLR level of IBD patients’ activity and remission period. The results showed that the NLR level of IBD patients and healthy people (**Figure 11**) Egger’s P=0.749 and Begg’s P=0.246, indicating no significant publication bias. There was no significant asymmetry in the shape of the funnel plot, and all studies were within 95%CI range. The activity level of IBD patients and the NLR level in remission (**Figure 12**) Egger’s P=0.254, Begg’s P=0.174 indicate no significant publication bias (P > 0.05). There was no significant asymmetry in the shape of the funnel plot, and all studies were within 95%CI range.

### 3.7 Subgroup analysis

To determine the source of heterogeneity, we performed a subgroup analysis. The results showed that high NLR was an important prognostic factor for the onset and progression of IBD, regardless of country, sample size, IBD type, study type, and diagnostic criteria. The subgroup analysis results were shown in **Table 3**.

### 3.8 Meta regression

We conducted a meta-regression analysis of IBD activity and NLR level in remission. **Table 4** shows univariate and multivariate meta-regression results. Univariate regression analysis results show that IBD patients, IBD type, study region and diagnostic criteria have no influence on patients’ NLR level. The results of multivariate analysis showed that the difference of study type and diagnostic criteria may be the source of heterogeneity (P < 0.05).

## 4 Discussion

Inflammatory bowel disease encompassing UC and CD, is a chronic inflammatory condition affecting the colorectal region, characterized by alternating periods of activity and remission. The assessment of disease activity and the prediction of treatment outcomes have become essential components of personalized management for patients with UC. Evaluating the activity of UC and CD involves a combination of clinical signs, laboratory indices, endoscopy, and histopathology. Currently, disease surveillance methods predominantly rely on colonoscopy, which is invasive, uncomfortable, time-consuming, and costly. This reliance may hinder patient compliance, and colonoscopy has been ranked as the least acceptable disease surveillance test among patients with CD (40, 41). Simple, non-invasive biomarkers are essential for mitigating the risks associated with invasive diagnostic procedures. Biomarkers previously studied for predicting inflammatory bowel disease (IBD) activity include serum markers such as C-reactive protein (CRP) and erythrocyte sedimentation rate (ESR), as well as fecal markers like calprotectin and lactoferrin. While CRP and ESR demonstrate high sensitivity in detecting IBD activity, their specificity is relatively low; these markers can be elevated not only due to IBD but also as a result of other extraintestinal inflammatory processes. Furthermore, genetic variability among individuals contributes to differences in CRP production, complicating accurate differentiation (42). The ESR is utilized less frequently than CRP due to its slower response to fluctuations in disease activity (43). Levels of the fecal marker calprotectin are proportional to neutrophil migration through the inflamed intestinal wall into the mucosa, and studies have demonstrated a correlation between calprotectin levels and endoscopic activity in patients with CD (44). Levels of fecal markers may be influenced by gut microbiota, as microbial diversity and quantity can affect marker expression and detection. Their routine use is further limited by high costs, lengthy sample processing times, and the challenges associated with fecal sample collection, which render them accessible to only a small proportion of patients with CD. Increasing evidence suggests that the pathology of IBD largely involves the progression of immunological lesions characterized by significant cellular infiltration, primarily by neutrophils, lymphocytes, macrophages, and plasma cells. Various systemic inflammatory markers, including the NLR, PLR, and LMR, derived from complete blood counts, have been reported as diagnostic and predictive indicators for IBD. While these markers are less costly and more easily accessible, their accuracy remains controversial. In response to this issue, we conducted a meta-analysis to evaluate the predictive value of peripheral blood inflammatory markers (NLR, PLR, and LMR) for IBD activity. The results showed that the level of NLR was significantly expressed in both UC and CD patients compared with the healthy population: (1) Compared with healthy individuals, the NLR levels of IBD patients were significantly elevated. The serum NLR level of IBD patients during the active phase was significantly higher than that during the remission phase. (2) The severity of IBD patients was positively correlated with the NLR and PLR levels. (3) There were no significant differences in serum LMR and LMR levels between IBD patients and healthy individuals, but the serum LMR level during the active phase of IBD patients was significantly lower than that during the remission phase. Finally, our study structure showed that NLR had better accuracy in diagnosing IBD AUC[ES=0.72,95%CI(0.69,0.75),P < 0.001].To address the high heterogeneity observed in the analyses, we conducted subgroup analyses and univariate/multivariate meta-regression analyses, which indicated that differences in study type and diagnostic criteria may contribute to this variability. The final results indicate that peripheral serum biomarkers such as the NLR and the PLR can serve as effective markers for predicting the activity of IBD. However, LMR levels did not demonstrate statistically significant differences between IBD patients and healthy individuals, nor among varying severities of IBD. Furthermore, LMR levels were significantly lower during the active phase of IBD compared to the remission phase, which introduces uncertainty and controversy regarding the predictive capability of LMR given these conflicting and non-significant results. These discrepancies may arise from limitations in the number of available studies; specifically, six studies reported differences in LMR levels between active and remission phases in IBD patients, while only two studies addressed the remaining two results. This limitation may have affected the outcomes of data pooling. We hope that future high-quality studies will further explore LMR levels at different stages of IBD, and we will continue to monitor research developments in this field.

The NLR was first identified as a marker of systemic inflammation in 2001 and has since been extensively studied in both malignant and non-malignant diseases (45). The NLR combines neutrophil and lymphocyte counts to provide a comprehensive view of the body’s immune-inflammatory status. Neutrophils are the first immune cells recruited to sites of inflammation, serving as the primary defense against pathogenic microorganisms and playing a crucial role in the innate immune system. In mucosal colon biopsies, the presence of neutrophils is associated with an increased risk of IBD recurrence and a poorer prognosis. Neutrophils are recruited from the circulatory system to areas of intestinal inflammation, where they eliminate pathogens by promoting inflammation through direct phagocytosis or by releasing NETs. These mechanisms contribute to mucosal healing and the resolution of inflammation (46, 47). Excessive neutrophil extracellular traps (NETs) can secrete inflammatory cytokines such as IL-1β and TNF-α, which amplify the inflammatory cascade and significantly contribute to the dysregulation of the immune-inflammatory response in the intestinal mucosa of patients with UC. Angelidou et al. (48) demonstrated that the activation of the REDD1/autophagy/NETs/IL-1β signaling pathway, which produces IL-1β, plays a crucial role in mediating intestinal inflammation and mucosal damage in UC. Furthermore, patients with IBD exhibit elevated levels of TNF-α, IL-1β, IL-16, and interferon-γ (IFN-γ) compared to healthy individuals (49, 50). When neutrophils (NEUs) are exposed to antigens, specific antigens bind to the ‘Fcγ receptor I/specific immunoglobulin G complex’ formed on the surface of sensitized NEUs. This binding stimulates the release of pro-inflammatory cytokines, such as TNF-α, which activates the protein kinase and NF-κB pathways. Ultimately, this activation leads to cellular differentiation, proliferation, and an increased production of pro-inflammatory cytokines, contributing to mucosal barrier defects in patients with UC (51, 52). The primary pathways for IL-1β production include the neutrophil serine protease pathway and the inflammasome-dependent caspase-1 pathway. In UC, IL-1β is produced both in the bloodstream and locally within the inflamed intestinal tract, contributing to tissue damage and inflammation (53). Conversely, neutrophils become “over-activated” in this pathological state. The increased expression of the anti-apoptotic protein A1 delays or reduces neutrophil apoptosis, significantly prolonging their lifespan and enhancing their activation. This over-activation results in the release of protein particles and reactive oxygen species, which disrupt the integrity of tight and adherens junctions, leading to dysfunction of the intestinal epithelial barrier. Such dysfunction further exacerbates tissue damage and results in typical UC mucosal manifestations, including cryptitis, mucosal erosion, and ulcer formation (54, 55). In addition, neutrophils upregulate pro-inflammatory chemokines, such as C-X-C motif chemokine ligand (CXCL) 1 (CXCL1), CXCL3, and CXCL8, which perpetuate the inflammatory cycle in colitis (56). CD inflammation can affect the entire intestinal tract, with the distal ileum being the most commonly involved site. Elevated levels of TNF-α, IL-1β, and IL-6 are also observed in patients with CD (57). TNF-α enhances the immune response of Th17 cells and promotes the secretion of cytokines such as interferon-γ (IFN-γ), while IL-6 facilitates the differentiation of Th17 cells, further mediating the destructive inflammatory response (58). The inflammatory response in Crohn’s disease (CD) is primarily sustained by the migration of Th17 lymphocytes and regulatory T (Treg) cells to the site of inflammation, mediated through interactions with integrins, such as α4β7 integrin, and other adhesion molecules, including leukocyte MacCAM-1 (59). Lymphocytes are crucial for antimicrobial defense and also play significant roles in organ development, tissue protection, regeneration, and mucosal homeostasis. Although these cells are components of the adaptive immune system, they originate from the same lymphoid progenitor cell population as other lymphocytes (60). Furthermore, lymphocytes are instrumental in the early phases of the immune response and in maintaining intestinal mucosal homeostasis by rapidly responding to cytokines and other signals produced by surrounding cells (61). Studies on Crohn’s disease and ulcerative colitis have identified lymphocyte dysfunction and abnormalities at both peripheral and mucosal levels, primarily characterized by a diminished response to mitogenic phytohemagglutinin and a tendency towards reduced absolute lymphocyte counts (31). The low lymphocyte counts observed in patients with active ulcerative colitis may stem from various factors, including mucosal lymphocyte infiltration, autoimmune-related apoptosis, malnutrition, and colonic bleeding or leakage. Platelets, which are essential for coagulation, are among the first cells recruited to the vascular endothelium at infection sites during both acute and chronic inflammation. They facilitate the recruitment of inflammatory cells by adhering to the endothelium or subendothelial space, regulating cell adhesion and extravasation, and activating monocytes, neutrophils, and endothelial cells. Furthermore, platelets synthesize and release substantial amounts of pro-inflammatory cytokines and chemokines (28, 62). During acute inflammation, cytokines such as IL-6 stimulate platelet differentiation, resulting in the release of activated platelets that contribute to both inflammation and thrombosis (30). Inflammatory mediators are released following intestinal microthrombosis in patients with IBD. Elevated platelet counts and activated platelets can trigger a series of inflammatory responses by increasing vascular permeability and promoting leukocyte migration, which exacerbates ischemia in the intestinal mucosa and may lead to irreversible intestinal damage (27). Additionally, platelets contain IL-8, which induces neutrophil aggregation and the release of superoxide; this process is mediated by adhesion molecules, resulting in platelet-neutrophil aggregation (30). Maugeri (63) found that platelets activate neutrophils through the release of HMGB1-containing platelet microparticles, which promotes the formation of an extracellular trapping network of neutrophils. Furthermore, monocytes can differentiate into macrophages and dendritic cells within tissues. In patients with active inflammatory bowel disease (IBD), elevated platelet counts result partly from an intestinal inflammatory response that promotes platelet maturation in the bone marrow. Concurrently, inflammatory factors drive continuous platelet consumption, which further stimulates platelet production and raises peripheral blood platelet counts. During inflammation, pro-inflammatory cytokines and chemokines stimulate monocyte production in the bone marrow and recruit these cells to sites of inflammation, where they differentiate into tissue-resident macrophages and dendritic cells. Consequently, sustained monocyte activation and a defective innate immune response play crucial roles in the pathogenesis of IBD (64). A substantial body of research indicates that elevated neutrophil and platelet counts, along with reduced lymphocyte counts, reflect the intensity of immunoinflammation in UC. Notably, elevated neutrophil levels can be observed even during clinical remission, provided that other infectious factors affecting leukocyte subtypes are excluded. Crispino et al. (65) reported that the NLR can be utilized in clinical practice to predict the response to anti-TNF-α therapy in patients with CD. Additionally, a recent study found that NLR and PLR levels can predict mucosal healing and the outcomes of anti-TNF-α monotherapy in patients with UC. These findings suggest that these markers could enhance the management of IBD in clinical settings (66).

This study represents the first meta-analysis to evaluate the predictive value of peripheral serum biomarkers, specifically the NLR, PLR, LMR, for Inflammatory Bowel Disease (IBD). The literature search conducted was comprehensive, and the statistical analysis applied was rigorous. However, our study is limited by a high degree of heterogeneity in the results. To address this limitation, we employed subgroup analysis and regression analysis to identify the sources of heterogeneity. The findings suggest that variations in study types and diagnostic criteria may contribute to this heterogeneity. (1) There were discrepancies in the definitions and assessment criteria for IBD activity among the original studies included. Currently, various tools are utilized in both clinical practice and research to define disease activity, and a globally accepted gold standard does not exist. The studies incorporated in this meta-analysis utilized diverse scoring systems, including the Mayo score, Lichtiger index, Pediatric Ulcerative Colitis Activity Index (PUCAI), Pediatric Crohn’s Disease Activity Index (PCDAI), Truelove & Witts criteria, Rachmilewitz index, and the Modified Truelove & Witts Severity Index (MTWSI).The clinical, endoscopic, and laboratory indicators included in each scale vary, as do the weighting and scoring criteria for the same indicators (such as the frequency of bloody stools and endoscopic findings) across different scales. Furthermore, these scales exhibit inconsistent definitions of remission and severity. For instance, a patient classified as ‘moderately active’ by the Mayo score may be categorized as ‘mild’ or ‘severe’ when evaluated using the Lichtiger Index or Rachmilewitz Index. This ‘tool diversity’ in defining and assessing disease activity leads to substantial differences in the inclusion criteria for study populations. Despite the significant heterogeneity observed in the studies, we found that all 23 included studies demonstrated that the NLR and PLR during the active phase were higher than those during the remission phase, or that NLR and PLR in IBD patients were elevated compared to healthy individuals (as illustrated in **Figures 2**, **4**, which show no cross-quadrant studies). This indicates that heterogeneity primarily affects the magnitude of the effect size rather than the direction of the conclusion. (2) The heterogeneity observed in this study may also be partially attributed to differences in the types of study designs included (prospective cohort studies versus retrospective cohort studies). Prospective studies, which adhere to predefined protocols, standardized data collection, and active follow-up, can more reliably assess baseline disease activity, control for confounding factors, and reduce bias. In contrast, retrospective studies rely on historical medical records and often encounter issues such as missing critical data, insufficient standardization, and incomplete follow-up, which directly impact the reliability and comparability of study results, thereby contributing to heterogeneity in meta-analyses.(3)There are notable differences in the cutoff values for NLR, PLR, and LMR across various regions. The observed range for NLR is between 1.91 and 3.6, while the range for PLR spans from 132.88 to 191.22. Studies employing lower cutoff values may categorize a greater proportion of patients into the ‘high exposure’ group, which could result in variations in the actual severity and composition of the ‘high NLR, PLR’ population across different studies. Furthermore, some studies did not specify their cutoff values. Given the limited number of studies and the fact that, although a few reported extreme values, most cutoff values were relatively concentrated within a specific range, we were unable to conduct subgroup analyses on cutoff values. Considering these factors, we anticipate that future high-quality prospective studies will further assess the predictive efficacy of NLR, PLR, and LMR for Inflammatory Bowel Disease (IBD), standardize diagnostic criteria, and narrow the range of cutoff values to mitigate bias.

## 5 Conclusion

In conclusion, we believe that NLR and PLR can be used as auxiliary indicators for clinical diagnosis of IBD and for assessing the activity of IBD. These indicators are simple to obtain, inexpensive, practical, have low operational difficulty, and have high patient compliance and clinical application value. Considering that LMR showed conflicting or insignificant results in several comparisons, LMR may not be a reliable independent marker. We still need more high-quality studies for further verification, which also provides a research direction for our subsequent studies.

## Data Availability

The original contributions presented in the study are included in the article/supplementary material. Further inquiries can be directed to the corresponding author.
