# Correction: The predictive role of peripheral serum inflammatory markers NLR, PLR, and LMR in ulcerative colitis and Crohn’s disease: a systematic review and meta-analysis

**DOI:** 10.3389/fimmu.2025.1676750

**Published:** 2025-08-13

**Authors:** Shufa Tan, Xiaoqing Yang, Xiaojing Mu, Shuang Liu, Yao Wang, Yuwei Li, Yuhong Bian, Chen Xu

**Affiliations:** ^1^ School of Integrative Medicine, Tianjin University of Traditional Chinese Medicine, Tianjin, China; ^2^ Tianjin Institute of Urology, the 2nd Hospital of Tianjin Medical University, Tianjin, China; ^3^ Department of Colorectal Surgery, Tianjin Union Medical Center, The First Affiliated Hospital of Nankai University, Tianjin, China

**Keywords:** inflammatory bowel disease, disease activity, NLR, PLR, LMR, meta-analysis

Author “Shufa Tan^1^” was erroneously omitted as equal contributing author. The author list has been corrected: authors should be:

“Shufa Tan1†, Xiaoqing Yang2†, Xiaojing Mu3†, Shuang Liu1, Yao Wang1, Yuwei Li3, Yuhong Bian1* and Chen Xu3*”

**^†^** These authors have contributed equally to this work

The original version of this article has been updated.

